# Inhaled Corticosteroids and COVID-19 Risk and Mortality: A Nationwide Cohort Study

**DOI:** 10.3390/jcm9113406

**Published:** 2020-10-23

**Authors:** Jae Chol Choi, Sun-Young Jung, Una A. Yoon, Seung-Hun You, Myo-Song Kim, Moon Seong Baek, Jae-Woo Jung, Won-Young Kim

**Affiliations:** 1 Department of Internal Medicine, Chung-Ang University Hospital, Chung-Ang University College of Medicine, Seoul 06973, Korea; medics27@cau.ac.kr (J.C.C.); wido21@cau.ac.kr (M.S.B.); jwjung@cau.ac.kr (J.-W.J.); 2 College of Pharmacy, Chung-Ang University, Seoul 06973, Korea; jsyoung@cau.ac.kr (S.-Y.J.); una.yoon@gmail.com (U.A.Y.); dbtmdgns23@naver.com (S.-H.Y.); myosong@cau.ac.kr (M.-S.K.)

**Keywords:** asthma, chronic obstructive pulmonary disease, COVID-19, SARS-CoV-2, steroids

## Abstract

Inhaled corticosteroids (ICS) could increase both the risk of coronavirus disease 2019 (COVID-19) and experiencing poor outcomes. To compare the clinical outcomes between ICS users and nonusers, COVID-19-related claims in the Korean Health Insurance Review and Assessment database were evaluated. To evaluate susceptibility to COVID-19 among patients with COPD or asthma, a nested case-control study was performed using the same database. In total, 7341 patients were confirmed to have COVID-19, including 114 ICS users and 7227 nonusers. Among 5910 patients who were hospitalized, death was observed for 9% of ICS users and 4% of nonusers. However, this association was not significant when adjusted for age, sex, region, comorbidities, and hospital type (aOR, 0.94; 95% CI, 0.43–2.07). The case-control analysis of COPD compared 640 cases with COVID-19 to 2560 matched controls without COVID-19, and the analysis of asthma compared 90 cases with COVID-19 to 360 matched controls without COVID-19. Use of ICS was not significantly associated with COVID-19 among patients with COPD (aOR, 1.02; 95% CI, 0.46–2.25) or asthma (aOR, 0.38; 95% CI, 0.13–1.17). Prior ICS use was not significantly associated with COVID-19 in patients with COPD or asthma, nor with clinical outcomes among patients with COVID-19.

## 1. Introduction

The current coronavirus disease 2019 (COVID-19) pandemic is caused by a novel coronavirus (severe acute respiratory syndrome coronavirus 2, SARS-CoV-2) [1]. Patients with COVID-19 and chronic respiratory diseases have relatively poor clinical outcomes [2]. Inhaled corticosteroids (ICS) combined with bronchodilators are broadly used for treating chronic obstructive pulmonary disease (COPD) and asthma [3,4]. Previous studies have indicated that there is no benefit in using systemic corticosteroids to treat SARS [5], and there are concerns regarding the use of ICS in COVID-19 cases, given the possibility of immunosuppression caused by long-term ICS treatment. Nevertheless, discontinuation of ICS treatment could lead to severe exacerbation in patients with asthma, and ICS treatment may even protect against viral infections, especially those involving SARS-CoV-2 [6,7].

There are limited clinical data regarding the potential benefits or harms associated with the use of ICS and other drugs for respiratory diseases among individuals who are at risk of contracting the SARS-CoV-2 infection or patients who have COVID-19. Several studies assessed the association between ICS use and the risk of hospitalization or mortality [8,9]. However, there was no potential control group of individuals who did not have COVID-19, which precludes a conclusion regarding the association between ICS use and the risk of COVID-19. Therefore, we performed this epidemiological study aiming to evaluate the potential benefits and harms associated with the use of ICS or other drugs for respiratory diseases among a large sample of individuals with and without COVID-19 who had detailed information regarding comorbidities and prior medication exposures.

## 2. Materials and Methods

### 2.1. Study Population and Data Sources

This nationwide population-based study evaluated de-identified records for individuals with and without COVID-19 from the Korean Health Insurance Review and Assessment (HIRA) database [10]. The database contains all COVID-19-related claim records for 234,427 individuals up to 15 May 2020 (see Methods in the Supplementary Materials). During the last 3 years, individuals’ records are linked to their healthcare utilization using finalized claims data (from January 2017 to 15 May 2020). The records are also linked to Korea Centers for Disease Control and Prevention data for confirming cases that involved COVID-19 and/or death. The data provides detailed information regarding demographics, diagnoses, prescriptions, procedures, and discharge outcomes. The diagnostic codes were assigned based on the Korean Classification of Diseases seventh revision, which is the Korean translation of the tenth revision of the International Classification of Diseases. All prescribed and dispensed medications were identified using Anatomical Therapeutic Chemical codes and HIRA general name codes.

The HIRA maintains the analysis dataset within a closed network and only shares a data schema that explains data parameters with participating researchers. The researchers are responsible for creating analysis codes using the data schema and uploading them to an online platform [10]. We have recently reported the associations between the prior use of renin-angiotensin-aldosterone system inhibitors and COVID-19-related outcomes using these databases [11]. The HIRA then runs the analysis codes for the applicable data within the closed network and subsequently provides the researchers with the resulting anonymized statistical data. The study protocol for analysis of de-identified patient data was exempted from review by the Institutional Review Board of Chung-Ang University (1041078-202005-HR-128-01).

The present study included all individuals who were ≥18 years old and identified as confirmed COVID-19 cases based on positive nasopharyngeal swab specimens that were tested using real-time reverse transcription-polymerase chain reaction assays [12]. The index date was defined as the date of the COVID-19 diagnosis. All individuals were followed until death or 15 May 2020.

### 2.2. Use of ICS and Other Drugs for Respiratory Diseases

The present study identified numerous drugs that were prescribed for respiratory diseases within 1 year before the index date. These drugs included ICS, long-acting β_2_ agonists, short-acting β_2_ agonists (SABAs), long-acting muscarinic antagonists, methylxanthines, leukotriene receptor antagonists (LTRAs), and phosphodiesterase 4 inhibitors. Table S1 describes the detailed types and codes for all respiratory disease-related drugs. If a combination of two different inhalers was used, each inhaler and drug(s) were counted separately. The present study defined respiratory disease-related drug users as individuals with continued drug use for ≥30 days during the 1-year period before the index date. Nonusers were defined as individuals who had never received drugs or had received them for < 30 days during the 1-year period before the index date. All doses for the ICS were converted to fluticasone equivalents according to the relevant guidelines [13], and the cumulative ICS dose was calculated during the 1-year period before the index date.

### 2.3. Data Collection and Definitions

Comorbidities were defined based on claim codes from within 1 year before the index date, which were used to determine the Charlson Comorbidity Index (CCI) (Table S2) [14]. The presence of COPD (J41–J44) and asthma (J45–J46) were identified based on the diagnostic codes and the presence of at least one drug treatment for respiratory disease or oral corticosteroid (OCS) treatment for ≥ 30 days within 1 year before the index date. Patients who fulfilled the criteria for both COPD and asthma were classified as COPD cases. The type of hospital that confirmed the COVID-19 diagnosis was defined according to the center’s number of beds and medical specialties. Data were also collected regarding previous use of OCS (Table S3), and OCS users were defined as individuals with a cumulative prednisolone-equivalent dose of ≥ 420 mg (15 mg daily for 4 weeks) during the previous year. Cumulative doses were calculated for OCS use during the 1-year period before the index date. Healthcare utilization was defined as COPD or asthma patients visiting an emergency room or being admitted to a hospital with a steroid medication prescription to account for acute exacerbation of disease. Data were extracted regarding antibiotics, antivirals, hydroxychloroquine, intravenous corticosteroids, and vasopressors that were used during the hospitalization for COVID-19. Procedure codes were also used to identify cases that involved conventional oxygen therapy, high flow nasal cannula, mechanical ventilation, extracorporeal membrane oxygenation (ECMO), and renal replacement therapy.

The primary study outcome was mortality until 15 May 2020. We also performed secondary analyses of the clinical outcomes among hospitalized COVID-19 patients, which included vasopressor use, modes of ventilation, ECMO, renal replacement therapy, and acute cardiac events (cardiac arrest, myocardial infarction, and acute heart failure).

### 2.4. Statistical Analysis

The ICS users and nonusers were compared in terms of their baseline demographic characteristics, comorbidities, exposure to drugs for respiratory diseases, and healthcare usages during the previous year. Hospitalized COVID-19 patients were also subjected to analyses of in-hospital treatments, procedures, and clinical outcomes according to ICS use. Logistic regression models were used to calculate odds ratios (ORs) and 95% confidence interval (CIs) for the different outcomes that were associated with the exposures of interest. The models separately included clinical outcomes (mortality and respiratory outcomes), drug exposures (listed above), ICS cumulative dose, COPD, and asthma. A composite variable was created to incorporate four categories of respiratory outcomes: conventional oxygen therapy, high flow nasal cannula, mechanical ventilation, and ECMO. Multivariate logistic regression analysis was performed to adjust for age, sex, region, CCI, and hospital type. A separate analysis was also performed to ascertain the effects of in-hospital procedures. The associations between ICS use and mortality were assessed with stratification according to age, sex, CCI, COPD, asthma, OCS use, and healthcare utilization.

To evaluate the susceptibility to COVID-19 among patients with COPD or asthma, a nested case-control study was performed using the HIRA database to examine the associations between a COVID-19 diagnosis and the use of ICS and other drugs for respiratory diseases. The cases were defined as patients with COVID-19 and prior COPD or asthma. For each case, up to four controls (COPD or asthma but no COVID-19) were randomly matched according to age, sex, region, and index date. The primary outcome was a COVID-19 diagnosis. The multivariable model did not include age, sex, and region as covariates, because the case-control matching was based on those variables.

For a sensitivity analysis, we assessed the potential effect of changes in the government’s treatment strategies for COVID-19. Up to 1 March 2020, all Korean patients with COVID-19 were hospitalized regardless of disease severity, although from 2 March and later only patients with moderate to severe COVID-19 were admitted to hospitals with negative pressure isolation rooms. Therefore, we performed analyses with stratification according to the COVID-19 diagnosis date (1 March 2020 and earlier vs. 2 March 2020 and later). All tests were two-tailed, and differences were considered statistically significant at *p*-values of< 0.05. All statistical analyses were performed using SAS software (version 9.4; SAS Institute, Cary, NC, USA).

## 3. Results

During the study period, 234,427 individuals had COVID-19-related claims, and 219,961 individuals (94%) were ≥ 18 years old. The main analysis included 7341 patients (3%) with confirmed COVID-19. The proportions of respiratory diseases were 9% for COPD (678/7341 patients) and 2% for asthma (123/7341 patients). The patients with confirmed COVID-19 were also classified as ICS users (114 patients) or nonusers (7227 patients).

The patients’ baseline characteristics are described in Table 1. Relative to nonusers, ICS users were older, more likely to have comorbidities, more likely to use healthcare within 1 year before the index date, and more likely to use OCS and other drugs for respiratory diseases. Among patients with COPD, ICS users were more likely to be older, have cardiovascular and cerebrovascular comorbidities, and be hospitalized during the previous year. The use of ICS was associated with elevated proportions of patients using OCS and other drugs for respiratory diseases, as well as a higher cumulative dose of OCS (Table S4). Among patients with asthma, the baseline characteristics of ICS users and nonusers were not significantly different (Table S5). A total of 5910 patients were hospitalized when the COVID-19 diagnosis was made, and these patients included 101 ICS users and 5809 nonusers. In-hospital treatments were more common among ICS users, although these findings were not observed in the subgroups of patients with COPD or asthma (Table S6).

The mortality rate for the entire cohort was 3% (227/7341 patients). Among hospitalized patients, the mortality rates were 9% among ICS users (9/101 patients) and 4% among nonusers (209/5809 patients) (Table S7). Higher proportions of conventional oxygen therapy and high flow nasal cannula were observed among ICS users. Similar findings were observed among patients with COPD, although there were no significant differences in the secondary outcomes between the ICS users and nonusers among patients with asthma (Table S7).

**Figure 1:**
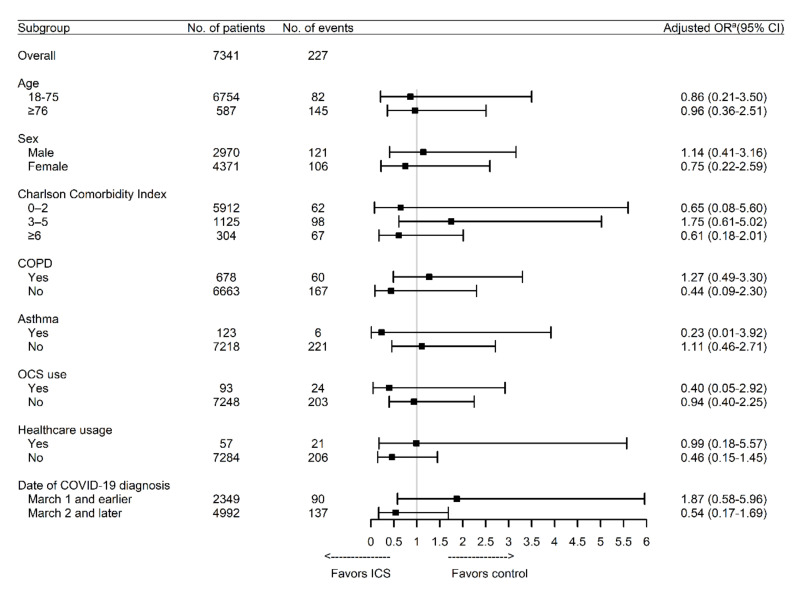
Association between the use of ICS and the risk of mortality according to subgroup. COPD: chronic obstructive pulmonary disease; COVID-19: coronavirus disease 2019; ICS: inhaled corticosteroids; OCS: oral corticosteroid. ^a^ Adjusted for age, sex, region, Charlson Comorbidity Index, and hospital type.

Table 2 shows the unadjusted and adjusted ORs from the logistic regression models. Use of ICS was associated with a significantly higher risk of mortality in the unadjusted analysis (OR, 3.11; 95% CI, 1.60–6.03; *p* < 0.001), although the association was not significant after adjustment for age, sex, region, CCI, and hospital type (adjusted OR, 0.94; 95% CI, 0.43–2.07; *p* = 0.88). This finding was not influenced when we additionally adjusted for in-hospital use of conventional oxygen therapy and high flow nasal cannula (Table S8). Similar associations were observed between the risk of mortality and other drugs for respiratory diseases, COPD, and asthma, although only OCS was independently associated with mortality. Similar to the primary analysis, ICS use was associated with a significantly higher risk of respiratory outcomes in the unadjusted analysis (OR, 2.99; 95% CI, 1.99–4.49; *p* < 0.001), but the association was not significant after adjustment for age, sex, region, CCI, and hospital type (adjusted OR, 1.35; 95% CI, 0.80–2.26; *p* = 0.26). Among the various drugs for respiratory diseases, use of methylxanthine and LTRA exhibited the strongest associations with a higher risk of respiratory outcomes in the adjusted analysis. Moreover, the adjusted risk of respiratory outcomes was increased among patients with COPD. However, assessment of the potential effect modification for ICS use according to subgroup revealed that no significant interactions were observed with any variable. The findings did not change substantially when the performed analyses were stratified according to the date of COVID-19 diagnosis (Figure 1).

The nested case-control analysis included 640 COPD patients with COVID-19 and 2560 matched controls with COPD but not COVID-19. Despite being matched according to age, sex, region, and index date, the controls had greater prevalence of various comorbidities, SABA and methylxanthine use, and a higher ICS cumulative dose (Table 3). The case-control analysis also included 90 asthma patients with COVID-19 and 360 matched controls with asthma but not COVID-19. There were generally no significant differences in the baseline characteristics of the cases and controls (Table 4). Use of ICS was not significantly associated with COVID-19 among patients with COPD (adjusted OR, 1.02; 95% CI, 0.46–2.25; *p* = 0.97), although ICS use was marginally associated with a lower risk of COVID-19 among patients with asthma (adjusted OR, 0.38; 95% CI, 0.13–1.17; *p* = 0.09) (Table 5).

## 4. Discussion

Among patients with COVID-19, the present study revealed no significant association between prior ICS use and mortality or respiratory outcomes after adjusting for baseline demographics and comorbidities. However, there was a significantly higher risk of mortality among patients with COVID-19 who were using OCS. A higher risk of respiratory outcomes was also observed among patients with COPD, relative to patients with asthma. Among patients with COPD, the use of ICS was not significantly associated with the risk of COVID-19, although ICS use tended to be associated with a reduced risk of COVID-19 among patients with asthma.

Older COVID-19 patients with comorbidities, including hypertension, diabetes, and cardiovascular and cerebrovascular diseases, develop severe illness more easily and have a poorer prognosis than patients without comorbidities [15,16]. However, the prevalence of chronic respiratory diseases, such as COPD and asthma, appears to be lower among patients with SARS and COVID-19 than among the general population [17,18]. Controversies exist regarding the associations between pre-existing chronic respiratory diseases and COVID-19. Preclinical models have suggested that impaired interferon production and other innate immune responses in both COPD and asthma could potentially lead to increased susceptibility to viral infection and the development of COVID-19 [19]. However, eosinophils play a central role in asthma and may promote viral clearance and antiviral host defense [20]. In addition, several type 2 cytokines have been shown to inhibit the secretion of proinflammatory cytokines and chemokines [21,22,23], which might counteract the “cytokine storms” of COVID-19. The expressions of angiotensin-converting enzyme 2 (ACE2), which is the entry receptor for SARS-CoV-2, and transmembrane protease serine 2 (TMPRSS2) may also affect susceptibility to SARS-CoV-2 infection. In a recent study of induced sputum samples from 330 participants, no significant difference in sputum ACE2 positivity was observed between patients with asthma and healthy subjects [24], which implies that the risk of COVID-19 may not be increased among patients with asthma. The prevalence of COPD and that of asthma in COVID-19 patients are similar to previously reported estimates of the Korean prevalence of COPD (8.8%) and that of asthma (3.9%) [25,26]. In this study, COPD or asthma status was not associated with COVID-19-related mortality, which conflicts with previous reports that chronic respiratory diseases were a significant risk factor for COVID-19-related mortality [2,27]. A recent report has suggested a relationship between the heterogeneity of asthma characteristics and the risk of potentially severe COVID-19 [28], although further studies are required to address this issue.

There are also a number of conflicting findings regarding ICS use during the COVID-19 pandemic. For example, ICS use for COPD and asthma was associated with an increased risk of pneumonia [29,30], although a preclinical study indicated that ICS use alone or in combination with bronchodilators inhibited human coronavirus 229E replication and proinflammatory cytokine production [6]. In patients with asthma, ICS use exhibited a dose-dependent association with reduced expressions of ACE2 and TMPRSS2 [24]. Furthermore, clinical improvement was observed in patients with COVID-19 who required oxygen after frequent and high-dose administration of inhaled ciclesonide [7]. The present study demonstrated that ICS users had a higher mortality rate and required more respiratory support than nonusers in the univariate analyses. However, the potential for confounding precludes causal inferences regarding the relationship between ICS use and COVID-19 severity. Even in patients with COPD, ICS use did not increase the risk of COVID-19. It is interesting that ICS use tended to decrease the risk of COVID-19 among patients with asthma, which is consistent with previous reports that ICS use might prevent SARS-CoV-2 infection or severe manifestation of COVID-19 [6,24]. Our analyses revealed that use of methylxanthines or LTRAs was associated with a higher risk of respiratory outcomes among patients with COVID-19. However, this unexpected finding may be related to unmeasured confounding and does not support the non-prescription of these drugs for COVID-19 patients with chronic respiratory diseases. Large-scale clinical trials and basic studies are needed to further clarify the role of respiratory disease drugs in modulating COVID-19 susceptibility and severity.

Systemic corticosteroids were extensively used during previous coronavirus outbreaks because of their anti-inflammatory effects, although previous studies regarding their use for SARS revealed no significant benefit [5]. A recent study of 17 million adults from a UK database revealed that a 10% higher risk of COVID-19-related mortality in asthma cases doubled to 20% in cases that additionally involved recent OCS use [27]. Before the release of the results of the RECOVERY trial [31], the World Health Organization did not recommend systemic corticosteroids in COVID-19 cases unless they were indicated for exacerbation of COPD or asthma [32]. However, these recommendations were mostly based on expert opinions and had caused uncertainty among COPD and asthma patients regarding whether a short course of low to moderate dose OCS could be used during the COVID-19 pandemic. The present study revealed that prehospital OCS use was associated with an increased risk of mortality among patients with COVID-19, even though the median daily dose of OCS in our study was relatively low (≤5 mg/d of prednisolone equivalents).

To assess the associations of chronic respiratory diseases with the clinical severity of COVID-19, we compared the adjusted ORs for respiratory outcomes, which consisted of various modes of ventilation and ECMO. The results revealed that both COPD and asthma were significantly associated with a higher risk of respiratory outcomes in the unadjusted analysis, although only the relationship with COPD remained significant after multivariable adjustment. In addition, patients with COPD had higher proportions of cardiovascular and cerebrovascular comorbidities and were more likely to receive respiratory support. Therefore, our findings suggest that COPD patients with COVID-19 should be closely monitored during hospitalization, especially patients with underlying comorbidities.

To the best of our knowledge, ours is one of the few epidemiological studies to assess whether prior use of ICS or other drugs for respiratory diseases was associated with outcomes in a nationwide cohort of COVID-19 patients. The main strength of our study is the use of nationwide claims data, which minimized the possibility of selection bias. Another strength of our study was the data regarding OCS use and COPD/asthma status, which were needed to adjust the treatment indication severity before the associations between prior drug use and clinical outcomes can be interpreted. At the time of publication, there has been a large observational study investigating the association between ICS and COVID-19-related death among patients with COPD or asthma using electronic health records of almost 1 million people in UK [33]. Although the study provides evidence that neither a benefit nor clear harm from ICS use against COVID-19 is demonstrated among these patients, our study in an Asian population is clinically relevant, given different prevalence of chronic respiratory diseases in different races/ethnicities.

The present study had several limitations. First, this retrospective study cannot exclude the possibility of residual confounding factors and precludes a causal inference regarding the relationships between use of drugs for respiratory diseases and the diagnosis and outcomes of COVID-19. Randomized controlled studies assessing the efficacy of ICS for COVID-19 treatment are underway, although these trials cannot evaluate the effects of prior ICS use among COVID-19 patients with chronic respiratory diseases. Second, the database did not include information regarding lung function, symptom severity using the Medical Research Council dyspnea scale, and smoking status, although matched or adjusted variables might be correlated with these factors. Third, patients who fulfilled the criteria for asthma-COPD overlap (ACO) were not classified in our analysis, and possible beneficial effects of ICS on the clinical outcomes of COVID-19 in these patients might have been overlooked. However, the definition of ACO is still controversial and may be ideally defined by means of treatable traits and biomarkers, which is not feasible in this medical claims-based study. Fourth, the low number of COPD and asthma patients treated with ICS and the high number of LTRA prescriptions in asthma patients may be unexpected and surprising. However, a recent study evaluating the effects of asthma and asthma medication on the clinical outcomes on COVID-19 using the same HIRA database identified similar prescription rate of ICS [34]. Moreover, the use of LTRAs was not associated with a higher risk of respiratory outcomes among COVID-19 patients with asthma (Table S9).Therefore, the possibility of changing the final conclusion by this limitation is not high. Fifth, we did not observe any meaningful dose–response effects for ICS due to limited sample size. Sixth, we only obtained information regarding drug prescriptions and could not evaluate actual drug consumption or prescription compliance. Seventh, this study cannot explain the mechanisms that might modify the risk of COVID-19 among COPD or asthma patients, or how ICS might influence the clinical outcomes of COVID-19. Additional studies are required to address these mechanisms. Finally, there is always a possibility of over-coding or under-coding, although the HIRA service vigorously audits insurance claims, and numerous peer-reviewed publications have used HIRA data.

## 5. Conclusions

In conclusion, our results suggest that prior use of ICS does not increase the risks of developing COVID-19, COVID-19-related mortality, or respiratory outcomes. However, clinicians should be aware of the possibility of clinical deterioration among patients who are systemic corticosteroid users, regardless of the dose or treatment duration. Until more information is available, during the current COVID-19 pandemic, there is no evidence to support the COVID-19-related discontinuation of ICS and other drugs for respiratory diseases among patients with COPD and asthma.

## Figures and Tables

**Table 1:** Baseline characteristics of patients with COVID-19 according to ICS use.

	All (*n* = 7341)	ICS Status		*p*
		Users (*n* = 114)	Nonusers (*n* = 7227)	
Age, years	47.1 (19.0)	57.4 (19.3)	46.9 (19.0)	<0.001
Male sex	2970 (40)	48 (42)	2922 (40)	0.72
Date of COVID-19 diagnosis				0.13
March 1 and earlier	2349 (32)	44 (39)	2305 (32)	
March 2 and later	4992 (68)	70 (61)	4922 (68)	
Comorbidities				
Diabetes	1299 (18)	38 (33)	1261 (17)	<0.001
Hypertension	1782 (24)	46 (40)	1736 (24)	<0.001
Myocardial infarction	71 (1)	3 (3)	68 (1)	0.10
Congestive heart failure	311 (4)	21 (18)	290 (4)	<0.001
Cerebrovascular disease	514 (7)	18 (16)	496 (7)	<0.001
COPD	678 (9)	52 (46)	626 (9)	<0.001
Asthma	123 (2)	61 (54)	62 (1)	<0.001
Chronic liver disease	1474 (20)	44 (39)	1430 (20)	<0.001
Chronic kidney disease	142 (2)	4 (4)	138 (2)	0.28
Malignancy	329 (4)	8 (7)	321 (4)	0.19
Charlson Comorbidity Index	1.4 (1.9)	3.3 (2.5)	1.3 (1.9)	<0.001
Other drugs for respiratory diseases				
LABA	121 (2)	110 (96)	11 (0.2)	<0.001
SABA	37 (1)	21 (18)	16 (0.2)	<0.001
LAMA	32 (0.4)	18 (16)	14 (0.2)	<0.001
Methylxanthine	110 (1)	37 (32)	73 (1)	<0.001
LTRA	149 (2)	52 (46)	97 (1)	<0.001
ICS use				
Cumulative dose, median (IQR), μg		15,000 (7500–45,000)	NA	
Total days of use, median (IQR)		90 (30–150)	NA	
OCS use	93 (1)	9 (8)	84 (1)	
Cumulative dose, median (IQR), mg	773 (605–1645)	980 (555–2180)	707 (605–1578)	0.41
Total days of use, median (IQR)	167 (121–373)	196 (111–332)	153 (121–376)	0.66
Healthcare usage				
Emergency room visit	24 (0.3)	8 (7)	16 (0.2)	<0.001
Hospitalization	54 (1)	15 (13)	39 (1)	<0.001

Data *n* (%) or mean (SD) unless indicated otherwise. *p*-values were calculated using Student’s *t*-test or the Mann–Whitney *U* test for continuous variables and using the chi-squared or Fisher’s exact test for categorical variables. COPD: chronic obstructive pulmonary disease; COVID-19: coronavirus disease 2019; ICS: inhaled corticosteroids; LABA: long-acting β_2_ agonist; LAMA: long-acting muscarinic antagonist; LTRA: leukotriene receptor antagonist; NA: not applicable; OCS: oral corticosteroid; SABA: short-acting β_2_ agonist.

**Table 2:** Risks of mortality and respiratory outcomes according to drug exposure, COPD, and asthma.

	No. of Patients	No. of Events	Unadjusted OR (95% CI)	*p*	Adjusted OR ^1^ (95% CI)	*p*
Mortality	7341	227				
ICS	114	10	3.11 (1.60–6.03)	<0.001	0.94 (0.43–2.07)	0.88
LABA	121	11	3.24 (1.72–6.12)	<0.001	0.85 (0.40–1.82)	0.67
SABA	37	3	2.79 (0.85–9.15)	0.09	0.87 (0.20–3.73)	0.85
LAMA	32	4	4.54 (1.58–13.05)	0.01	0.69 (0.20–2.32)	0.54
Methylxanthine	110	20	7.54 (4.56–12.48)	<0.001	1.45 (0.78–2.69)	0.24
LTRA	149	15	3.69 (2.13–6.39)	<0.001	1.36 (0.70–2.64)	0.37
OCS use	93	24	12.07 (7.43–19.61)	<0.001	3.42 (1.89–6.17)	<0.001
ICS cumulative dose						
<15,000 μg	60	4	2.31 (0.83–6.42)	0.81	1.17 (0.34–3.98)	0.71
≥15,000 μg	54	6	4.04 (1.71–9.54)	0.053	0.83 (0.31–2.24)	0.65
COPD	678	60	3.78 (2.78–5.13)	<0.001	0.95 (0.65–1.39)	0.78
Asthma	123	6	1.62 (0.71–3.73)	0.25	0.92 (0.35–2.42)	0.86
Respiratory outcomes ^2^	7341	934				
ICS	114	34	2.99 (1.99–4.49)	<0.001	1.35 (0.80–2.26)	0.26
LABA	121	36	2.98 (2.01–4.43)	<0.001	1.20 (0.72–1.99)	0.48
SABA	37	11	2.93 (1.44–5.94)	0.003	1.72 (0.65–4.55)	0.28
LAMA	32	14	5.40 (2.68–10.90)	<0.001	1.14 (0.49–2.64)	0.76
Methylxanthine	110	49	5.76 (3.93–8.44)	<0.001	1.81 (1.13–2.92)	0.01
LTRA	149	44	2.97 (2.07–4.25)	<0.001	1.58 (1.004–2.48)	0.048
OCS use	93	38	4.90 (3.22–7.45)	<0.001	1.52 (0.90–2.54)	0.11
ICS cumulative dose						
<15,000 μg	60	16	2.56 (1.44–4.55)	0.34	2.02 (0.97–4.22)	0.08
≥15,000 μg	54	18	3.52 (1.99–6.22)	0.02	0.96 (0.47–1.93)	0.32
COPD	678	188	3.04 (2.53–3.66)	<0.001	1.31 (1.03–1.67)	0.03
Asthma	123	25	1.77 (1.14–2.76)	0.01	1.20 (0.69–2.10)	0.52

COPD: chronic obstructive pulmonary disease; ICS: inhaled corticosteroids; LABA: long-acting β_2_ agonist; LAMA: long-acting muscarinic antagonist; LTRA: leukotriene receptor antagonist; OCS: oral corticosteroid; SABA: short-acting β_2_ agonist. ^1^ Adjusted for age, sex, region, Charlson Comorbidity Index, and hospital type. ^2^ A composite variable that included conventional oxygen therapy, high flow nasal cannula, mechanical ventilation, and extracorporeal membrane oxygenation.

**Table 3:** Baseline characteristics of COPD patients with COVID-19 and matched controls.

	All (*n* = 3200)	COVID-19 (*n* = 640)	Non-COVID-19 (*n* = 2560)	*p*
Age, years	58.4 (19.7)	58.4 (19.7)	58.4 (19.7)	MV
Male sex	1370 (43)	274 (43)	1096 (43)	MV
Date of COVID-19 diagnosis				
March 1 and earlier	944 (30)	198 (31)	NA	
March 2 and later	2256 (71)	442 (69)	NA	
Comorbidities				
Diabetes	1120 (35)	213 (33)	907 (35)	0.31
Hypertension	1477 (46)	283 (44)	1194 (47)	0.27
Myocardial infarction	108 (3)	13 (2)	95 (4)	0.04
Congestive heart failure	506 (16)	86 (13)	420 (16)	0.07
Cerebrovascular disease	666 (21)	111 (17)	555 (22)	0.02
Chronic liver disease	1279 (40)	222 (35)	1057 (41)	0.002
Chronic kidney disease	272 (9)	27 (4)	245 (10)	<0.001
Malignancy	527 (16)	66 (10)	461 (18)	<0.001
Charlson Comorbidity Index	3.8 (3.1)	3.3 (2.4)	4.1 (3.1)	<0.001
Other drugs for respiratory diseases				
ICS	286 (9)	50 (8)	236 (9)	0.26
LABA	368 (12)	61 (10)	307 (12)	0.08
SABA	138 (4)	15 (2)	123 (5)	0.01
LAMA	206 (6)	31 (5)	175 (7)	0.07
Methylxanthine	444 (14)	67 (10)	377 (15)	0.01
LTRA	289 (9)	55 (9)	234 (9)	0.67
PDE4 inhibitor	2 (0.1)	0	2 (0.1)	>0.99
ICS use				
Cumulative dose, median (IQR), μg	30,000 (12,000–69,000)	19,500 (9000–48,000)	33,000 (12,000–73,500)	0.04
Total days of use, median (IQR)	120 (30–210)	120 (30–210)	120 (30–210)	0.22
OCS use	176 (6)	22 (3)	154 (6)	
Cumulative dose, median (IQR), mg	1183 (613–1835)	689 (605–1682)	1200 (680–1855)	0.27
Total days of use, median (IQR)	251 (125–393)	185 (121–398)	255 (141–392)	0.26
Healthcare usage				
Emergency room visit	141 (4)	20 (3)	121 (5)	0.08
Hospitalization	284 (9)	48 (8)	236 (9)	0.17

Data *n* (%) or mean (SD) unless indicated otherwise. *p*-values were calculated using Student’s *t*-test or the Mann–Whitney *U* test for continuous variables and using the chi-squared or Fisher’s exact test for categorical variables. COPD: chronic obstructive pulmonary disease; COVID-19: coronavirus disease 2019; ICS: inhaled corticosteroids; LABA: long-acting β_2_ agonist; LAMA: long-acting muscarinic antagonist; LTRA: leukotriene receptor antagonist; MV: matching variable; NA: not applicable; OCS: oral corticosteroid; PDE4: phosphodiesterase 4; SABA: short-acting β_2_ agonist.

**Table 4:** Baseline characteristics of asthma patients with COVID-19 and matched controls.

	All (*n* = 450)	COVID-19 (*n* = 90)	Non-COVID-19 (*n* = 360)	*p*
Age, years	52.8 (17.3)	52.7 (17.5)	52.9 (17.3)	MV
Male sex	170 (38)	34 (38)	136 (38)	MV
Date of COVID-19 diagnosis				
March 1 and earlier	120 (27)	23 (26)	NA	
March 2 and later	330 (73)	67 (74)	NA	
Comorbidities				
Diabetes	117 (26)	25 (28)	92 (26)	0.67
Hypertension	158 (35)	28 (31)	130 (36)	0.37
Myocardial infarction	8 (2)	1 (1)	7 (2)	>0.99
Congestive heart failure	43 (10)	6 (7)	37 (10)	0.30
Cerebrovascular disease	58 (13)	6 (7)	52 (14)	0.049
Chronic liver disease	143 (32)	32 (36)	111 (31)	0.39
Chronic kidney disease	29 (6)	3 (3)	26 (7)	0.18
Malignancy	49 (11)	8 (9)	41 (11)	0.50
Charlson Comorbidity Index	3.0 (2.4)	2.7 (1.9)	3.1 (2.5)	0.07
Other drugs for respiratory diseases				
ICS	219 (49)	43 (48)	176 (49)	0.85
LABA	205 (46)	41 (46)	164 (46)	>0.99
SABA	87 (19)	15 (17)	72 (20)	0.47
LAMA	1 (0.2)	0	1 (0.3)	>0.99
Methylxanthine	101 (22)	21 (23)	80 (22)	0.82
LTRA	197 (44)	40 (44)	157 (44)	0.89
ICS use				
Cumulative dose, median (IQR), μg	18,000 (12,000–45,000)	18,000 (12,000–45,000)	18,000 (12,000–45,000)	0.54
Total days of use, median (IQR)	60 (30–150)	90 (30–180)	60 (30–120)	0.89
OCS use	51 (11)	9 (10)	42 (12)	
Cumulative dose, median (IQR), mg	820 (570–1671)	980 (555–1375)	815 (600–1671)	0.66
Total days of use, median (IQR)	162 (114–318)	196 (111–275)	159 (118–318)	0.67
Healthcare usage				
Emergency room visit	13 (3)	1 (1)	12 (3)	0.48
Hospitalization	25 (6)	1 (1)	24 (7)	0.04

Data *n* (%) or mean (SD) unless indicated otherwise. *p*-values were calculated using Student’s *t*-test or the Mann–Whitney *U* test for continuous variables and using the chi-squared or Fisher’s exact test for categorical variables. COVID-19: coronavirus disease 2019; ICS: inhaled corticosteroids; LABA: long-acting β_2_ agonist; LAMA: long-acting muscarinic antagonist; LTRA: leukotriene receptor antagonist; MV: matching variable; NA: not applicable; OCS: oral corticosteroid; SABA: short-acting β_2_ agonist.

**Table 5:** Risk of COVID-19 according to drug exposures among patients with COPD and asthma.

	No. of Patients	No. of Events	Unadjusted OR (95% CI)	*p*	Adjusted OR ^1^ (95% CI)	*p*
**COPD**	3200	640				
ICS	286	50	0.84 (0.61–1.15)	0.27	1.02 (0.46–2.25)	0.97
LABA	368	61	0.77 (0.58–1.03)	0.08	0.94 (0.45–1.98)	0.87
SABA	138	15	0.48 (0.28–0.82)	0.01	0.52 (0.13–2.17)	0.37
LAMA	206	31	0.69 (0.47–1.03)	0.07	0.47 (0.11–1.94)	0.30
Methylxanthine	444	67	0.68 (0.51–0.89)	0.01	0.72 (0.33–1.58)	0.41
LTRA	289	55	0.94 (0.69–1.27)	0.67	1.20 (0.57–2.53)	0.64
OCS use	176	22	0.56 (0.35–0.88)	0.01	0.89 (0.28–2.91)	0.85
ICS cumulative dose						
<15,000 μg	115	24	1.04 (0.66–1.64)	0.40	2.24 (0.94–5.37)	0.02
≥15,000 μg	171	26	0.71 (0.46–1.08)	0.13	0.24 (0.03–1.73)	0.07
**Asthma**	450	90				
ICS	219	43	0.96 (0.60–1.52)	0.85	0.38 (0.13–1.17)	0.09
LABA	205	41	1.00 (0.63–1.59)	>0.99	0.48 (0.16–1.44)	0.19
SABA	87	15	0.80 (0.43–1.48)	0.47	0.85 (0.23–3.12)	0.80
LAMA	1	0		0.99		0.99
Methylxanthine	101	21	1.07 (0.62–1.84)	0.82	0.79 (0.17–3.72)	0.77
LTRA	197	40	1.03 (0.65–1.65)	0.89	1.42 (0.50–4.04)	0.51
OCS use	51	9	0.84 (0.39–1.80)	0.66	0.87 (0.11–7.24)	0.90
ICS cumulative dose						
<15,000 μg	107	20	0.90 (0.50–1.61)	0.70	0.16 (0.02–1.26)	0.13
≥15,000 μg	112	23	1.01 (0.58–1.77)	0.82	0.60 (0.18–2.03)	0.57

COPD: chronic obstructive pulmonary disease; COVID-19: coronavirus disease 2019; ICS: inhaled corticosteroids; LABA: long-acting β_2_ agonist; LAMA: long-acting muscarinic antagonist; LTRA: leukotriene receptor antagonist; OCS: oral corticosteroid; SABA: short-acting β_2_ agonist. ^1^ Adjusted for Charlson Comorbidity Index and hospital type.

## Supplementary Materials

The following are available online at https://www.mdpi.com/2077-0383/9/11/3406/s1. Methods: Coronavirus disease 2019 (COVID-19)-related claims data. Table S1: Types and codes for drugs used to treat respiratory diseases. Table S2: Comorbidities based on the Charlson Comorbidity Index. Table S3: Types and codes for oral corticosteroid. Table S4: Baseline characteristics of COPD patients with COVID-19 according to ICS use. Table S5: Baseline characteristics of asthma patients with COVID-19 according to ICS use. Table S6: Treatments for hospitalized patients with COVID-19. Table S7: Clinical outcomes among hospitalized COVID-19 patients. Table S8: Risk of mortality according to drug exposure, COPD, and asthma. Table S9: Risk of respiratory outcomes according to drug exposures among patients with asthma.
