# Supplementary material for: Inhaled Corticosteroids and COVID-19 Risk and Mortality: A Nationwide Cohort Study
Source: J Clin Med. 2020 Oct 23;9(11):3406. doi: 10.3390/jcm9113406 (PMC7690894; doi:10.3390/jcm9113406)
Supplement: Supplementary file 1 [file jcm-09-03406-s001.pdf]

Methods

Coronavirus disease 2019 (COVID-19)-related claims data

Claims were identified based on the following codes: MT043 (national disaster fundholding type 3/02), D6584 (real-time reverse transcription-polymerase chain reaction assays for COVID-19), B342 (coronavirus infection, unspecified site), B972 (coronavirus as the cause of diseases classified to other chapters), Z208 (contact with and exposure to other communicable diseases), Z290 (isolation), U18 (provisional assignment of new diseases of uncertain etiology or emergency use), U181 (novel coronavirus infection), Z038 (observation for other suspected diseases and conditions), Z115 (special screening examination for other viral diseases), and U071/072 (COVID-19). Furthermore, claims were reviewed for other COVID-19-related fee codes regarding admission, management, intensive care, isolation, community treatment center, screening clinic, and use of a negative pressure room.

Table S1. Types and codes for drugs used to treat respiratory diseases.

	Type	ATC	HIRA general name code
**ICS**			
	budesonide 20 mg	R03BA02	119530CSI
	budesonide 40 mg	R03BA02	119531CSI
	fluticasone 6 mg	R03BA05	162233CSI
	fluticasone 15 mg	R03BA05	162235CSI
	fluticasone 30 mg	R03BA05	162236CSI
	fluticasone 6 mg	R03BA05	162232CSI
	fluticasone 3 mg	R03BA05	500431CSI
	fluticasone 6 mg	R03BA05	500432CSI
	ciclesonide 9.6 mg	R03BA08	497131CSI
	ciclesonide 4.8 mg	R03BA08	497130CSI
**ICS/LABA**			
	beclomethasone 12 mg/formoterol 0.72 mg	R03AK08	544200CSI
	budesonide 9.6 mg/formoterol 0.27 mg	R03AK07	543800CSI
	budesonide 19.2 mg/formoterol 0.54 mg	R03AK07	543900CSI
	budesonide 19.2 mg/formoterol 0.54 mg	R03AK07	544100CSI
	budesonide 4.8 mg/formoterol 0.27 mg	R03AK07	544000CSI
	budesonide 9.6 mg/formoterol 0.27 mg	R03AK07	801100CSI
	budesonide 18 mg/salmeterol 1.5 mg	R03AK12	681000CSI
	budesonide 9 mg/salmeterol 1.5 mg	R03AK12	681100CSI
	fluticasone 15 mg/formoterol 0.6 mg	R03AK11	542800CSI
	fluticasone 30 mg/formoterol 1.2 mg	R03AK11	543000CSI
	fluticasone 6 mg/formoterol 0.6 mg	R03AK11	542900CSI
	fluticasone 3 mg/vilanterol 0.75 mg	R03AK10	636700CSI
	fluticasone 6 mg/vilanterol 0.75 mg	R03AK10	636800CSI
	fluticasone 15 mg/salmeterol 3 mg	R03AK06	543400CSI
	fluticasone 6 mg/salmeterol 3 mg	R03AK06	543100CSI
	fluticasone 15 mg/salmeterol 3 mg	R03AK06	543300CSI
	fluticasone 30 mg/salmeterol 3 mg	R03AK06	543600CSI
	fluticasone 7 mg/salmeterol 1.4 mg	R03AK06	544400CSI
	fluticasone 30 mg/salmeterol 3 mg	R03AK06	543500CSI
	fluticasone 6 mg/salmeterol 3 mg	C09CA01	543200CSI
**LABA**			
	procaterol 2 mg	R03AC16	218330CSI
	indacaterol 4.5 mg	R03AC18	611901CSI
	indacaterol 9 mg	R03AC18	611902CSI
**LABA/LAMA**			
	aclidinium 24 mg/formoterol 0.72 mg	R03AL05	635300CSI
	glycopyrronium 1.5 mg/indacaterol 3.3 mg	R03AL04	800100CSI
	olodaterol 0.15 mg/tiotropium 0.15 mg	R03AL06	643700CSI
	umeclidinium 1.875 mg/vilanterol 0.75 mg	R03AL03	631200CSI
**SABA**			
	salbutamol 20 mg	R03AC02	225531CSI
**LAMA**			
	tiotropium 0.54 mg	R03BB04	457330CSI
	tiotropium 0.54 mg	R03BB04	457301CCH

	tiotropium 0.15 mg	R03BB04	503430CSI
	umeclidinium 2.226 mg	R03BB07	641101CSI
	aclidinium 24 mg	R03BB05	633730CSI
	glycopyrronium 1.5 mg	R03BB06	635903CSI
**Methylxanthine**			
	theophylline 40 g	R03DA04	237031ASY
	theophylline 100 mg	R03DA04	237001ACR
	theophylline 200 mg	R03DA04	237003ACR
	theophylline 200 mg	R03DA04	237003ATR
	theophylline 400 mg	R03DA04	237005ATR
	theophylline 130 mg	R03DA04	237002ACR
	theophylline 200 mg	R03DA04	237003ACH
	theophylline 200 mg	R03DA04	237030ASY
	aminophylline 100 mg	R03DA05	107301ATB
	aminophylline 100 mg	R03DA05	107301ATR
	aminophylline 225 mg	R03DA05	107303ATR
	bamiphylline 300 mg	R03DA08	113801ATB
	acebrophylline 100 mg	R03DA04	100701ACH
	acebrophylline 200 mg	R03DA04	100702ATR
	doxofylline 400 mg	R03DA11	439101ATB
	theobromine 300 mg	R03DA07	503301ACH
**LTRA**			
	pranlukast 50 mg	R03DC02	216430ASY
	pranlukast 70 mg	R03DC02	216431ASY
	pranlukast 100 mg	R03DC02	216432ASY
	pranlukast 10 g	R03DC02	216433ASY
	pranlukast 112.5 mg	R03DC02	216401ACH
	pranlukast 100 mg	R03DC02	216402ASS
	pranlukast 112.5 mg	R03DC02	216403ACH
	pranlukast 75 mg	R03DC02	216404ATB
	pranlukast 50 mg	R03DC02	216405ASS
	pranlukast 70 mg	R03DC02	216406ASS
	pranlukast 140 mg	R03DC02	216407ASS
	pranlukast 50 mg	R03DC02	216408ATB
	montelukast 5 mg	R03DC03	374601ATB
	montelukast 5 mg	R03DC03	374601ATD
	montelukast 10 mg	R03DC03	374602ATB
	montelukast 10 mg	R03DC03	374602ATD
	montelukast 4 mg	R03DC03	374603AGN
	montelukast 4 mg	R03DC03	374603ATB
	montelukast 4 mg	R03DC03	374603ATD
	montelukast 10 mg/levocetirizine 5 mg	R03DC03	659900ACH
	montelukast 5 mg/levocetirizine 5 mg	R03DC03	670600ATB
	ibudilast 10 mg	R03DC04	172701ACH
**PDE4 inhibitor**			
	roflumilast 0.5 mg	R03DX07	614701ATB

ATC: Anatomical Therapeutic Chemical identifier; HIRA: Health Insurance Review and Assessment database; ICS: inhaled corticosteroids; LABA: long-acting β_2 agonist; LAMA: long-acting muscarinic antagonist; LTRA: leukotriene receptor antagonist; PDE4: phosphodiesterase 4; SABA: short-acting β_2 agonist.

Table S2. Comorbidities based on the Charlson Comorbidity Index.

	**ICD-10 codes**
**Charlson Comorbidity Index**	
Myocardial infarction	I21, I22, I252
Congestive heart failure	I099, I110, I130, I132, I255, I420, I425, I426, I427, I428, I429, I43, I50, P290
Peripheral vascular disease	I70, I71, I731, I738, I739, I771, I790, I792, K551, K558, K559, Z958, Z959
Cerebrovascular disease	G45, G46, I60, I61, I62, I63, I64, I65, I66, I67, I68, I69, H340
Dementia	F00, F01, F02, F03, G30, F051, G311
Chronic pulmonary disease	I278, I279, J41, J42, J43, J44, J45, J46, J47, J60, J61, J62, J63, J64, J65, J66, J67, J684, J701, J703
Rheumatic disease	M05, M06, M315, M32, M33, M34, M351, M353, M360
Peptic ulcer disease	K25, K26, K27, K28
Mild liver disease	B18, K700, K701, K702, K703, K709, K713, K714, K715, K717, K73, K74, K760, K762, K763, K764, K768, K769, Z944
Moderate or severe liver disease	I850, I859, I864, I982, K704, K711, K721, K729, K765, K766, K767
Diabetes without complications	E100, E101, E106, E108, E109, E110, E111, E116, E118, E119, E120, E121, E126, E128, E129, E130, E131, E136, E138, E139, E140, E141, E146, E148, E149
Diabetes with complications	E102, E103, E104, E105, E107, E112, E113, E114, E115, E117, E122, E123, E124, E125, E127, E132, E133, E134, E135, E137, E142, E143, E144, E145, E147
Paraplegia and hemiplegia	G041, G114, G800, G81, G82, G830, G831, G832, G833, G834, G839
Renal disease	I120, I131, N030, N031, N032, N033, N034, N035, N036, N037, N038, N039, N050, N051, N052, N053, N054, N055, N056, N057, N058, N059, N18, N19, N250, Z490, Z491, Z492, Z940, Z992
Any malignancy	C00, C01, C02, C03, C04, C05, C06, C07, C08, C09, C10, C11, C12, C13, C14, C15, C16, C17, C18, C19, C20, C21, C22, C23, C24, C25, C26, C30, C31, C32, C33, C34, C37, C38, C39, C40, C41, C43, C45, C46, C47, C48, C49, C50, C51, C52, C53, C54, C55, C56, C57, C58, C60, C61, C62, C63, C64, C65, C66, C67, C68, C69, C70, C71, C72, C73, C74, C75, C76, C81, C82, C83, C84, C85, C88, C90, C91, C92, C93, C94, C95, C96, C97
Metastatic solid tumor	C77, C78, C79, C80
AIDS/HIV	B20, B21, B22, B24
Hypertension, uncomplicated [1]	I10
Hypertension, complicated [1]	I11, I12, I13, I15

ICD: International Classification of Diseases; AIDS: acquired immune deficiency syndrome; HIV: human immunodeficiency virus. [1] Hypertension is not included in the Charlson comorbidities and was identified separately using the ICD-10 codes.

Table S3. Types and codes for oral corticosteroid.

	Type	ATC	HIRA general name code
Deflazacort	deflazacort 6 mg	H02AB13	140801ATB
Dexamethasone	dexamethasone 0.5 mg	H02AB02	141901ATB
	dexamethasone 0.75 mg	H02AB02	141903ATB
Betamethasone	betamethasone 0.25 mg + d-chlorpheniramine 2 mg	H02AB01	296900ATB
	betamethasone 0.5 mg	H02AB01	116401ATB
Hydrocortisone	hydrocortisone 10 mg	H02AB09	170901ATB
	hydrocortisone 5 mg	H02AB09	170906ATB
Methylprednisolone	methylprednisolone 4 mg	H02AB04	193302ATB
	methylprednisolone 1 mg	H02AB04	193305ATB
Prednisolone	prednisolone 5 mg	H02AB06	217001ATB
Triamcinolone	triamcinolone 1 mg	H02AB08	243201ATB
	triamcinolone 2 mg	H02AB08	243202ATB
	triamcinolone 4 mg	H02AB08	243203ATB
Fludrocortisone	fludrocortisone 100 μg	H02AA02	160201ATB

ATC: Anatomical Therapeutic Chemical identifier; HIRA: Health Insurance Review and Assessment database.

Table S4. Baseline characteristics of COPD patients with COVID-19 according to ICS use.

		ICS status		
	**All (n = 678)**	**Users (n = 52)**	**Nonusers (n = 626)**	***p***
Age, years	58.2 (19.3)	65.6 (17.1)	57.5 (19.3)	0.004
Male sex	276 (41)	24 (46)	252 (40)	0.41
Date of COVID-19 diagnosis				0.99
March 1 and earlier	235 (35)	18 (35)	217 (35)	
March 2 and later	443 (65)	34 (65)	409 (65)	
Comorbidities				
Diabetes	222 (33)	22 (42)	200 (32)	0.13
Hypertension	290 (43)	28 (54)	262 (42)	0.09
Myocardial infarction	13 (2)	3 (6)	10 (2)	0.07
Congestive heart failure	89 (13)	17 (33)	72 (12)	<0.001
Cerebrovascular disease	115 (17)	14 (27)	101 (16)	0.046
Chronic liver disease	235 (35)	23 (44)	212 (34)	0.13
Chronic kidney disease	28 (4)	3 (6)	25 (4)	0.47
Malignancy	68 (10)	3 (6)	65 (10)	0.29
Charlson Comorbidity Index	3.2 (2.4)	4.0 (2.5)	3.2 (2.3)	0.01
Other drugs for respiratory diseases				
LABA	62 (9)	51 (98)	11 (2)	<0.001
SABA	15 (2)	10 (19)	5 (1)	<0.001
LAMA	31 (5)	17 (33)	14 (2)	<0.001
Methylxanthine	68 (10)	25 (48)	43 (7)	<0.001
LTRA	56 (8)	29 (56)	27 (4)	<0.001
ICS use				
Cumulative dose, median (IQR), μg		19500 (10500–46500)	NA	
Total days of use, median (IQR)		90 (30–150)	NA	
OCS use	22 (3)	6 (12)	16 (3)	
Cumulative dose, median (IQR), mg	689 (605–1682)	980 (555–2180)	707 (605–1578)	0.04
Total days of use, median (IQR)	185 (121–398)	196 (111–332)	153 (121–276)	0.39
Healthcare usage				
Emergency room visit	21 (3)	6 (12)	15 (2)	0.003
Hospitalization	50 (7)	13 (25)	37 (6)	<0.001

Data n (%) or mean (SD) unless indicated otherwise. *P*-values were calculated using Student's *t*-test or the Mann-Whitney *U* test for continuous variables and using the chi-squared or Fisher's exact test for categorical variables. COPD: chronic obstructive pulmonary disease; COVID-19: coronavirus disease 2019; ICS: inhaled corticosteroids; LABA: long-acting β_2 agonist; LAMA: long-acting muscarinic antagonist; LTRA: leukotriene receptor antagonist; NA: not applicable; OCS: oral corticosteroid; SABA: short-acting β_2 agonist.

Table S5. Baseline characteristics of asthma patients with COVID-19 according to ICS use.

	All	ICS status		
		**Users**	**Nonusers**	
	**(n = 123)**	**(n = 61)**	**(n = 62)**	***p***
Age, years	52.6 (18.8)	50.0 (18.2)	55.0 (18.1)	0.13
Male sex	44 (36)	23 (38)	21 (34)	0.66
Date of COVID-19 diagnosis				0.80
March 1 and earlier	49 (40)	25 (41)	24 (39)	
March 2 and later	74 (60)	36 (59)	38 (61)	
Comorbidities				
Diabetes	31 (25)	15 (25)	16 (26)	0.88
Hypertension	37 (30)	18 (30)	19 (31)	0.89
Myocardial infarction	2 (2)	0	2 (3)	0.50
Congestive heart failure	7 (6)	4 (7)	3 (5)	0.72
Cerebrovascular disease	9 (7)	4 (7)	5 (8)	>0.99
Chronic liver disease	39 (32)	20 (33)	19 (31)	0.80
Chronic kidney disease	3 (2)	1 (2)	2 (3)	>0.99
Malignancy	10 (8)	4 (7)	6 (10)	0.74
Charlson Comorbidity Index	2.6 (1.9)	2.5 (1.8)	2.8 (1.9)	0.29
Other drugs for respiratory diseases				
LABA	58 (47)	58 (95)	0	<0.001
SABA	18 (15)	10 (16)	8 (13)	0.58
LAMA	0	0	0	
Methylxanthine	29 (24)	12 (20)	17 (27)	0.31
LTRA	56 (46)	23 (38)	33 (53)	0.08
ICS use				
Cumulative dose, median (IQR), μg		15000 (6000–39500)	NA	
Total days of use, median (IQR)		60 (30–150)	NA	
OCS use	12 (10)	3 (5)	9 (15)	
Cumulative dose, median (IQR), mg	908 (580–1423)	835 (555–980)	1250 (605–1470)	0.37
Total days of use, median (IQR)	182 (116–285)	167 (111–196)	250 (121–294)	0.37
Healthcare usage				
Emergency room visit	2 (2)	2 (3)	0	0.24
Hospitalization	3 (2)	2 (3)	1 (2)	0.62

Data n (%) or mean (SD) unless indicated otherwise. *P*-values were calculated using Student's *t*-test or the Mann-Whitney *U* test for continuous variables and using the chi-squared or Fisher's exact test for categorical variables. COVID-19: coronavirus disease 2019; ICS: inhaled corticosteroids; LABA: long-acting β_2 agonist; LAMA: long-acting muscarinic antagonist; LTRA: leukotriene receptor antagonist; NA: not applicable; OCS: oral corticosteroid; SABA: short-acting β_2 agonist.

Table S6. Treatments for hospitalized patients with COVID-19.

	All	ICS status		p
		Users	Nonusers	
**All patients**	n = 5910	n = 101	n = 5809	
Antibiotics	2574 (44)	61 (60)	2513 (43)	<0.001
Antivirals	2664 (45)	58 (57)	2606 (45)	0.01
Lopinavir/ritonavir	2644 (45)	58 (57)	2586 (45)	0.01
Darunavir	58 (1)	0	58 (1)	0.63
Interferon	63 (1)	2 (2)	61 (1)	0.29
Oseltamivir	12 (0.2)	1 (1)	11 (0.2)	0.19
Hydroxychloroquine	2068 (35)	42 (42)	2026 (35)	0.16
Steroids [1]	272 (5)	10 (10)	262 (5)	0.03
**COPD patients**	n = 585	n = 45	n = 540	
Antibiotics	347 (59)	29 (64)	318 (59)	0.47
Antivirals	330 (56)	29 (64)	301 (56)	0.26
Lopinavir/ritonavir	325 (56)	29 (64)	296 (55)	0.21
Darunavir	11 (2)	0	11 (2)	>0.99
Interferon	15 (3)	1 (2)	14 (3)	>0.99
Oseltamivir	1 (0.2)	1 (2)	0	0.08
Hydroxychloroquine	264 (45)	21 (47)	243 (45)	0.83
Steroids [1]	59 (10)	6 (13)	53 (10)	0.44
**Asthma patients**	n = 107	n = 55	n = 52	
Antibiotics	58 (54)	31 (56)	27 (52)	0.65
Antivirals	56 (52)	28 (51)	28 (54)	0.76
Lopinavir/ritonavir	56 (52)	28 (51)	28 (54)	0.76
Darunavir	0	0	0	
Interferon	1 (1)	1 (2)	0	>0.99
Oseltamivir	0	0	0	
Hydroxychloroquine	40 (37)	20 (36)	20 (38)	0.82
Steroids [1]	8 (7)	4 (7)	4 (8)	>0.99

Data n (%). *P*-values were calculated using the chi-squared or Fisher's exact test, as appropriate. COVID-19: coronavirus disease 2019; ICS: inhaled corticosteroids. [1] Only intravenous methylprednisolone or hydrocortisone.

Table S7. Clinical outcomes among hospitalized COVID-19 patients.

		ICS status		
	**All**	**Users**	**Nonusers**	***p***
**All patients**	n = 5910	n = 101	n = 5809	
In-hospital mortality	218 (4)	9 (9)	209 (4)	0.01
Vasopressor use	187 (3)	4 (4)	183 (3)	0.56
Conventional oxygen therapy	907 (15)	33 (33)	874 (15)	<0.001
High flow nasal cannula	182 (3)	9 (9)	173 (3)	0.004
Mechanical ventilation	127 (2)	2 (2)	125 (2)	>0.99
ECMO	21 (0.4)	1 (1)	20 (0.3)	0.30
Renal replacement therapy	30 (1)	0	30 (0.5)	>0.99
Acute cardiac events				
Cardiac arrest	43 (1)	2 (2)	41 (1)	0.17
Myocardial infarction	257 (4)	6 (6)	251 (4)	0.45
Acute heart failure	377 (6)	6 (6)	371 (6)	0.86
**COPD patients**	n = 585	n = 45	n = 540	
In-hospital mortality	58 (10)	7 (16)	51 (9)	0.19
Vasopressor use	38 (1)	3 (3)	35 (1)	>0.99
Conventional oxygen therapy	179 (31)	22 (49)	157 (29)	0.01
High flow nasal cannula	45 (8)	7 (16)	38 (7)	0.07
Mechanical ventilation	23 (4)	1 (2)	22 (4)	>0.99
ECMO	1 (0.2)	0	1 (0.2)	>0.99
Renal replacement therapy	3 (1)	0	3 (1)	>0.99
Acute cardiac events				
Cardiac arrest	12 (2)	2 (4)	10 (2)	0.23
Myocardial infarction	28 (5)	3 (7)	25 (5)	0.47
Acute heart failure	57 (10)	4 (9)	53 (10)	>0.99
**Asthma patients**	n = 107	n = 55	n = 52	
In-hospital mortality	6 (6)	2 (4)	4 (8)	0.43
Vasopressor use	3 (3)	1 (2)	2 (4)	0.61
Conventional oxygen therapy	25 (23)	11 (20)	14 (27)	0.40
High flow nasal cannula	4 (4)	2 (4)	2 (4)	>0.99
Mechanical ventilation	2 (2)	1 (2)	1 (2)	>0.99
ECMO	2 (2)	1 (2)	1 (2)	>0.99
Renal replacement therapy	0	0	0	
Acute cardiac events				
Cardiac arrest	1 (1)	0	1 (2)	0.49
Myocardial infarction	5 (5)	3 (5)	2 (4)	>0.99
Acute heart failure	7 (7)	2 (4)	5 (10)	0.26

Data n (%). *P*-values were calculated using the chi-squared or Fisher's exact test, as appropriate. COPD: chronic obstructive pulmonary disease; COVID-19: coronavirus disease 2019; ECMO: extracorporeal membrane oxygenation; ICS: inhaled corticosteroids.

Table S8. Risk of mortality according to drug exposure, COPD, and asthma.

	No. of patients	No. of events	Unadjusted OR (95% CI)	*p*	Adjusted OR [1] (95% CI)	*p*
Mortality	7341	227				
ICS	114	10	3.11 (1.60–6.03)	<0.001	0.79 (0.33–1.93)	0.61
LABA	121	11	3.24 (1.72–6.12)	<0.001	0.71 (0.30–1.66)	0.43
SABA	37	3	2.79 (0.85–9.15)	0.09	1.01 (0.24–4.26)	0.99
LAMA	32	4	4.54 (1.58–13.05)	0.01	0.54 (0.15–1.96)	0.35
Methylxanthine	110	20	7.54 (4.56–12.48)	<0.001	1.26 (0.65–2.43)	0.50
LTRA	149	15	3.69 (2.13–6.39)	<0.001	1.06 (0.50–2.26)	0.89
OCS use	93	24	12.07 (7.43–19.61)	<0.001	3.88 (1.99–7.58)	<0.001
ICS cumulative dose						
<15000 μg	60	4	2.31 (0.83–6.42)	0.81	1.37 (0.39–4.89)	0.38
≥15000 μg	54	6	4.04 (1.71–9.54)	0.053	0.55 (0.17–1.73)	0.25
COPD	678	60	3.78 (2.78–5.13)	<0.001	0.83 (0.55–1.26)	0.39
Asthma	123	6	1.62 (0.71–3.73)	0.25	0.95 (0.34–2.71)	0.93

COPD: chronic obstructive pulmonary disease; ICS: inhaled corticosteroids; LABA: long-acting β_2 agonist; LAMA: long-acting muscarinic antagonist; LTRA: leukotriene receptor antagonist; OCS: oral corticosteroid; SABA: short-acting β_2 agonist. [1] Adjusted for age, sex, region, Charlson Comorbidity Index, hospital type, conventional oxygen therapy, and high flow nasal cannula.

Table S9. Risk of respiratory outcomes according to drug exposures among patients with asthma.

	No. of patients	No. of events	Unadjusted OR (95% CI)	*p*	Adjusted OR [1] (95% CI)	*p*
**Asthma**	123	25				
ICS	61	11	0.75 (0.31–1.83)	0.53	0.76 (0.25–2.30)	0.62
LABA	58	11	0.85 (0.35–2.06)	0.72	0.85 (0.28–2.58)	0.77
SABA	18	3	0.76 (0.20–2.84)	0.68	3.20 (0.47–21.80)	0.23
LAMA	0	0				
Methylxanthine	29	10	2.77 (1.08–7.12)	0.03	1.86 (0.58–6.02)	0.30
LTRA	56	12	1.13 (0.47–2.73)	0.78	1.13 (0.37–3.45)	0.83
OCS use	12	1	0.33 (0.04–2.68)	0.30		0.95
ICS cumulative dose						
<15000 μg	34	7	0.89 (0.32–2.47)	0.79	2.12 (0.53–8.47)	0.04
≥15000 μg	27	4	0.60 (0.18–2.01)	0.45	0.18 (0.03–1.08)	0.02

ICS: inhaled corticosteroids; LABA: long-acting β_2 agonist; LAMA: long-acting muscarinic antagonist; LTRA: leukotriene receptor antagonist; OCS: oral corticosteroid; SABA: short-acting β_2 agonist. [1] Adjusted for age, sex, region, Charlson Comorbidity Index, and hospital type.
